# Functional outcomes and rehabilitation behaviors following ankle sprain in amateur padel players: a cross-sectional study

**DOI:** 10.3389/fmed.2026.1887372

**Published:** 2026-07-27

**Authors:** Faris Alanazi, Hussain Saad Al Dera, Thamer Al Shammary, AlHassan AlSharif, Abdulrahman Ahmed, Ahmad Al Mansour

**Affiliations:** 1 King Abdullah International Medical Research Center, Riyadh, Saudi Arabia; 2 Department of Rehabilitation, Ministry of the National Guard - Health Affairs, Riyadh, Saudi Arabia; 3 King Saud Bin Abdulaziz University for Health Sciences, Riyadh, Saudi Arabia; 4 Department of Rehabilitation, Prince Mohammed Bin Nasser Hospital, Jazan Health Cluster, Jazan, Saudi Arabia

**Keywords:** ankle sprain, cross-sectional studies, padel, rehabilitation, risk factors, sports injuries

## Abstract

**Introduction:**

Ankle sprain is one of the most common injuries in racket sports; however, data on its prevalence, risk factors, and functional impact among amateur padel players remain limited. To determine the prevalence and clinical profile of ankle sprain injuries, identify associated risk factors, and evaluate the relationship between preventive behaviors and functional outcomes among amateur padel players in Saudi Arabia.

**Methods:**

A cross-sectional study was conducted among 187 amateur padel players. Data on demographics, playing exposure, injury characteristics, and preventive behaviors were collected. Functional outcomes were assessed using the Foot and Ankle Disability Index (FADI). Parametric statistical analyses, including independent-samples *t*-tests, one-way ANOVA, Pearson correlation, and multivariable logistic regression, were performed.

**Results:**

The prevalence of ankle sprain was 14.97% (95% CI: 10.30–20.80). Injuries were predominantly right-sided (60.71%) and commonly caused by landing (57.14%), with most classified as moderate (46.43%). Functional limitation was reported by 75.00%, and recurrent sprains occurred in 42.86%. Mean FADI score was 86.12 ± 8.74. Playing experience ≥1 year (aOR = 3.18, *p* = 0.021) and frequency ≥2 sessions/week (aOR = 2.88, *p* = 0.026) were independent predictors of injury. Preventive behaviors were not associated with injury risk but were significantly associated with higher FADI scores, particularly balance and strengthening exercises (Δ = 8.20, *p* = 0.008).

**Conclusion:**

Ankle sprains are common among amateur padel players and are associated with persistent functional impairment. Playing exposure was the primary factor associated with injury risk, while preventive behaviors play a significant role in improving functional recovery.

## 1 Introduction

Ankle sprain is among the most prevalent musculoskeletal injuries in sports involving rapid changes in direction, jumping, and dynamic lower-limb loading, and it represents a substantial clinical burden due to its high recurrence rates and potential for long-term functional impairment (1). In racket sports, including tennis, squash, and, more recently, padel, the combination of multidirectional movements, abrupt deceleration, and repeated landing tasks places considerable stress on the lateral ankle complex (2). Although often perceived as minor injuries, ankle sprains are frequently associated with residual symptoms such as instability, reduced proprioception, and impaired performance, which may persist despite early return to sport (3). These factors contribute to the development of chronic ankle instability and highlight the importance of understanding both injury occurrence and post-injury functional outcomes in athletic populations (3). Recent advances in computational biomechanics and data-driven modeling have further improved the understanding of ligament loading mechanisms and fatigue failure associated with ankle injuries (4). Deep learning integrated with computational ligament mechanics has provided novel insights into injury risk prediction and ligament failure mechanisms, complementing traditional epidemiological and clinical research on sports-related ankle sprains (5).

Emerging evidence has begun to characterize injury patterns in padel, a rapidly growing sport worldwide (6). Previous studies have reported that lower-limb injuries, particularly those involving the ankle, are common due to the sport's specific biomechanical and environmental demands (7). Ferreira et al. (8) and Altun et al. (9) have demonstrated that the high frequency of lateral displacements and reactive movements in padel increases susceptibility to injury, particularly during landing and directional changes. Additionally, research across broader sports populations has identified playing exposure, including training frequency and cumulative experience, as key determinants of injury risk (10). In parallel, preventive strategies such as neuromuscular training, balance exercises, and external support have been shown to improve functional outcomes and reduce residual deficits following ankle sprain (11). However, the extent to which these factors influence injury occurrence vs. recovery in amateur padel players remains insufficiently characterized (11).

Despite growing interest in padel-related injuries, there is still a need for a comprehensive evaluation of their prevalence, risk factors, and functional consequences within a single cohort. This need is particularly important among amateur players, who may differ from elite athletes in their training load, injury management, and use of preventive practices (6). Existing literature has largely focused on injury incidence or general patterns, with limited emphasis on functional outcomes and the role of modifiable behaviors in recovery (1). Furthermore, the potential divergence between factors associated with injury risk and those influencing post-injury function has not been adequately explored. Addressing these gaps is clinically important for developing targeted strategies that not only reduce the occurrence of injury but also optimize rehabilitation and long-term functional performance.

Accordingly, the present study aimed to determine the prevalence and clinical profile of ankle sprain injuries among amateur padel players in Saudi Arabia, to identify associated demographic, clinical, and playing-exposure risk factors, and to evaluate the relationship between preventive behaviors and functional outcomes. It was hypothesized that increased playing exposure would be independently associated with a higher likelihood of ankle sprain, whereas preventive behaviors would be more strongly associated with improved functional outcomes than with reduced injury occurrence.

## 2 Material and methods

### 2.1 Study design, ethics, and settings

A cross-sectional study was conducted between 01/04/2025 and 30/03/2026 among amateur padel players in Saudi Arabia, using an online survey. Ethical approval was obtained from the Institutional Review Board of King Abdullah International Medical Research Center (KAIMRC), Riyadh, Saudi Arabia (Protocol No. NRR25/086/8; IRB approved), and written informed consent was obtained from all participants prior to participation. The study was conducted in accordance with the ethical principles outlined in the Declaration of Helsinki (12).

### 2.2 Sample size calculation

The sample size was estimated a priori using G^*^Power software (version 3.1.9.7, Heinrich Heine University, Düsseldorf, Germany) based on the primary objective of identifying associations between ankle sprain occurrence and categorical risk factors using chi-square tests of independence in a cross-sectional design (13). Assuming a moderate effect size (Cohen's w = 0.30), which is commonly reported in sports injury epidemiology for behavioral and exposure-related factors, a two-tailed alpha level of 0.05, statistical power of 80%, and degrees of freedom = 2–3 depending on the variable categories (13), the minimum required sample size was estimated to be 143 participants. The final achieved sample included 187 participants, exceeding the minimum requirement and thereby increasing statistical power to approximately 88–90%, enhancing the reliability of the findings and allowing for more stable subgroup analyses.

### 2.3 Participants

Participants were recruited via convenience sampling through an online survey distributed via social media platforms and networks commonly accessed by amateur padel players in Saudi Arabia, as described in the study protocol. Eligibility was determined using self-reported data collected via a structured questionnaire that included items on playing history and ankle injury status. Participants were required to be aged 15 years or older and actively engaged in padel at an amateur level. Ankle sprain status was identified based on participant self-report of a previous ankle sprain sustained during padel play, supported by descriptive information on injury mechanism, symptoms, and recurrence (14). Individuals who did not play padel, were below the specified age threshold, or provided incomplete or inconsistent responses were excluded from the analysis.

All submitted responses were reviewed for completeness and internal consistency prior to inclusion. Baseline data collected at the time of survey completion included demographic characteristics, playing exposure variables (experience and frequency), injury history, and preventive behaviors. Participants who met the eligibility criteria and provided complete datasets were included in the final analysis. The structured questionnaire was developed based on previously published sports injury surveillance tools and relevant literature. Specifically, the questionnaire was informed by published sports injury surveillance recommendations, epidemiological studies of padel-related injuries, and the validated Foot and Ankle Disability Index (FADI), which was used to assess functional outcomes (14). These sources were used to guide the selection of variables, injury definitions, and questionnaire structure. Before implementation, the questionnaire underwent content validation by a panel comprising sports physiotherapists, sports medicine clinicians, and academic researchers with expertise in musculoskeletal injuries and sports epidemiology. Their feedback was used to refine the wording, relevance, and clarity of the questionnaire items. The revised questionnaire was subsequently pilot-tested in a small group of amateur padel players to assess comprehensibility and feasibility, and minor editorial modifications were made before the final version was administered. Because the questionnaire incorporated previously validated instruments and established injury definitions, formal construct validity testing was not performed for the newly assembled survey.

### 2.4 Ankle sprain occurrence

The primary outcome variable was the occurrence of ankle sprain among amateur padel players. This was operationally defined as a self-reported history of at least one ankle sprain sustained during padel participation (6). Consistent with epidemiological definitions used in sports injury research, an ankle sprain was described to participants as an injury involving stretching or tearing of the ankle ligaments, accompanied by symptoms including pain, swelling, and functional limitations (6). Data were obtained through a structured online questionnaire in which participants indicated whether they had experienced an ankle sprain, the number of episodes, and the timing of the most recent injury. This self-reported approach is widely used in cross-sectional sports injury studies where clinical verification is not feasible, and it has been shown to provide acceptable validity when supported by symptom-based descriptors and contextual injury information. Participants were asked to report injuries that occurred within the preceding 12 months to capture relevant injury events while maintaining a defined recall period.

### 2.5 Functional status (FADI Score)

Functional outcome was assessed using the FADI (15), a validated patient-reported outcome measure commonly used to evaluate functional limitations associated with ankle disorders. The FADI consists of 26 items covering activities of daily living and sports-related function, each scored on a 5-point Likert scale ranging from 0 (unable to perform) to 4 (no difficulty) (16). The total score ranges from 0 to 104, with higher scores indicating better functional status. Participants with a history of ankle sprain completed the FADI as part of the questionnaire. For interpretability, functional status was categorized as mild (90–99), moderate (70–89), or severe (<70) disability, based on previously reported thresholds (16). The FADI has demonstrated strong reliability, internal consistency, and responsiveness in populations with ankle instability and sports-related injuries, supporting its suitability for this study. The FADI questionnaire was administered in its validated Arabic version, where applicable, or in English for participants proficient in English (17). Standardized instructions were provided within the survey interface to ensure consistent interpretation of items.

### 2.6 Injury characteristics and clinical profile

Detailed injury-related variables were collected to characterize the clinical profile of ankle sprains (18). These included injury laterality (right, left, bilateral), mechanism of injury (e.g., landing, collision, sudden directional change), severity grade (mild, moderate, severe), time loss from sport (in days), pain intensity (measured using a numerical rating scale from 0 to 10), presence of swelling, recurrence of sprain, and associated injuries (knee, hip/groin, lower back) (18). Functional limitation was defined as self-reported difficulty in performing usual daily or sports-related activities following the ankle injury (yes/no). Recurrent sprain was defined as the occurrence of two or more ankle sprain episodes within the same 12-month recall period. This variable was analyzed separately from episodes of giving way, which reflect subjective instability rather than confirmed reinjury. Time loss from sport and return-to-play duration were both assessed; time loss referred to the period of complete absence from sport participation, whereas return-to-play duration captured the total time until the resumption of padel activity, which may include modified participation. Injury severity was classified as Grade I (mild), Grade II (moderate), or Grade III (severe) based on participant-reported symptoms, including pain intensity, swelling, functional limitations, and time lost from sport, consistent with standard ligament injury grading frameworks (19). Additional functional indicators were assessed to capture residual ankle instability and neuromuscular deficits. Perceived instability was defined as a subjective sensation of the ankle “giving way” or lack of joint stability during activity. Episodes of giving way were quantified as the number of self-reported instability events occurring within the past 6 months (19). Balance and strength deficits were assessed using participant self-report (yes/no) of perceived impairments in postural control and ankle muscle strength during daily or sports activities (19).

### 2.7 Exposure variables: playing experience and frequency

Playing exposure variables were assessed as key independent predictors of ankle sprain. Playing experience was defined as the duration of engagement in padel and categorized as <1 year, 1–3 years, and >3 years (10). Playing frequency was defined as the number of sessions per week and categorized as 1, 2–3, or≥4 sessions. These variables were self-reported and reflect cumulative and weekly exposure to sport-specific loading, respectively. In line with prior epidemiological studies, exposure was further dichotomized (e.g., <1 year vs. ≥1 year; 1 session vs. ≥2 sessions/week) for regression analyses to evaluate their association with injury risk (10).

### 2.8 Preventive behaviors

Preventive behaviors were evaluated as modifiable factors that may influence injury risk and functional outcomes. These included warm-up practices (always, sometimes, never), use of external ankle support (always, sometimes, never), and engagement in ankle-specific balance and strengthening exercises (yes, sometimes, no). Data were collected via self-report using predefined categorical responses. These behaviors were operationalized based on established injury-prevention frameworks, in which neuromuscular training and external support are recognized as key components of ankle injury management. This approach was based on the International Olympic Committee consensus statement on methods for recording and reporting epidemiological data on injury and illness in sport and the Clinical Practice Guideline of the Orthopedic Section of the American Physical Therapy Association for lateral ankle ligament sprains, both of which emphasize neuromuscular exercise and external ankle support as evidence-based injury-prevention strategies (20). For analytical purposes, categories were grouped, where appropriate (consistent vs. inconsistent use), to assess their associations with both injury occurrence and functional outcomes. For regression analyses, preventive behavior variables were dichotomized into optimal (always performing warm-up, consistent use of ankle support, regular engagement in exercises) vs. suboptimal (sometimes/never) categories to facilitate interpretation of effect estimates.

### 2.9 Footwear and playing surface

Environmental and equipment-related variables included footwear type and playing surface. Footwear was categorized as running shoes, casual sneakers, padel-specific shoes, or other, with padel-specific shoes serving as the reference category due to their sport-specific design features. The playing surface was classified as artificial turf or other surfaces. These variables were self-reported and included based on their potential influence on traction, stability, and injury risk, as highlighted in prior sports biomechanics and injury prevention research.

### 2.10 Covariates: demographic and clinical characteristics

Baseline covariates included age (years), sex (male/female), body mass index (BMI, kg/m^2^), smoking status (yes/no), and presence of chronic medical conditions (yes/no). Chronic medical conditions were defined as self-reported physician-diagnosed long-term health conditions (e.g., diabetes, hypertension, asthma) that may influence physical activity or injury risk. Age and BMI were analyzed as continuous variables, while categorical variables were coded as binary or nominal as appropriate. These data were collected through the questionnaire and included to control for potential confounding effects in the analysis, as they have been previously associated with injury susceptibility and recovery outcomes in sports populations.

### 2.11 Statistical analysis

Data were analyzed using IBM SPSS Statistics version 24 (IBM Corp., Armonk, NY, USA). Continuous variables were assessed for normality and are presented as mean ± standard deviation, while categorical variables are summarized as frequencies and percentages. The prevalence of ankle sprain was calculated with corresponding 95% confidence intervals and evaluated using a one-sample proportion test. Comparisons between participants with and without ankle sprain were performed using independent-samples *t*-tests for continuous variables and Pearson's chi-square or Fisher's exact tests for categorical variables, as appropriate. Differences in functional outcomes (FADI scores) across preventive behavior categories were assessed using independent-samples *t*-tests or one-way analysis of variance (ANOVA), with effect sizes reported where applicable. Pearson correlation analysis was also conducted to examine the relationship between exercise engagement and functional scores. Multivariable logistic regression analysis was performed to identify independent predictors of ankle sprain, with adjusted odds ratios (aORs) and 95% confidence intervals reported after controlling for demographic, clinical, and exposure-related variables. All statistical tests were two-tailed, and a *p*-value of <0.05 was considered statistically significant. Variables included in the multivariable logistic regression model were selected based on clinical relevance and a univariable screening threshold of *p* < 0.20. Multicollinearity was assessed using variance inflation factors (VIF), with a threshold of <5 considered acceptable. Model fit was evaluated using the Hosmer–Lemeshow goodness-of-fit test and pseudo-R^2^ statistics (Cox & Snell and Nagelkerke R^2^). Missing data were minimal (<5%) and handled using a complete-case analysis, with only participants who provided complete responses included in each respective analysis.

## 3 Results

The study population consisted predominantly of young adult male amateur padel players with moderate playing exposure and limited use of preventive strategies (Table 1). The mean age was 29.84 ± 6.12 years with a mean BMI of 25.73 ± 3.84 kg/m^2^, and males comprised 78.61% of participants. More than half of the cohort were relatively inexperienced (<1 year: 53.48%) and played infrequently (1 session/week: 65.24%), primarily on artificial turf (84.49%). Non-specific footwear was commonly used, with only 19.25% reporting padel-specific shoes. Preventive behaviors were suboptimal, as only 44.39% consistently performed warm-ups, while ankle support use was notably low (always: 2.67%; never: 79.14%). Additionally, nearly half of the participants (49.73%) reported not engaging in ankle-strengthening exercises, indicating a substantial gap in injury-prevention practices.

**Table 1:** Demographic, clinical, and playing characteristics of amateur padel players in Saudi Arabia, presented in analytical format (*N* = 187).

Variable	Category/Comparison	*n*	Value
Age (years)	Mean ± SD	187	29.84 ± 6.12
BMI (kg/m^2^)	Mean ± SD	187	25.73 ± 3.84
Gender	Male	147	78.61%
	Female	40	21.39%
Smoking status	Yes	47	25.13%
	No	140	74.87%
Chronic condition	Yes	101	54.01%
	No	86	45.99%
Padel experience	<1 year	100	53.48%
	1–3 years	67	35.83%
	>3 years	20	10.70%
Playing frequency/week	1 session	122	65.24%
	2–3 sessions	45	24.06%
	≥4 sessions	20	10.70%
Playing surface	Artificial turf	158	84.49%
	Other surfaces	29	15.51%
Footwear type	Running shoes	73	39.04%
	Casual sneakers	56	29.95%
	Padel-specific shoes	36	19.25%
	Other	22	11.76%
Warm-up behavior	Always	83	44.39%
	Sometimes	85	45.45%
	Never	19	10.16%
Ankle support use	Always	5	2.67%
	Sometimes	34	18.18%
	Never	148	79.14%
Ankle exercises	Yes	43	22.99%
	Sometimes	51	27.27%
	No	93	49.73%

BMI, Body Mass Index; SD, Standard Deviation; n, Number of participants.

Ankle sprain injuries were observed in 14.97% of participants (95% CI: 10.30–20.80), indicating a moderate prevalence within the cohort (Table 2). Injuries were predominantly unilateral, affecting the right side in 60.71% of cases, and were most commonly caused by landing-related mechanisms (57.14%). The majority of injuries were classified as self-reported perceived moderate severity (46.43%), followed by self-reported perceived mild severity (39.29%), with relatively short time loss from sport (3.12 ± 1.95 days) despite notable symptom burden, including pain (4.86 ± 1.72) and swelling (67.86%). Functional limitation was reported by 75.00% of injured players, and recurrent sprains occurred in 42.86%, highlighting persistent impairment. Importantly, none of the injured participants sought medical attention (100.00%), underscoring a critical gap in injury management.

**Table 2:** Prevalence and comprehensive clinical profile of ankle sprain injuries among amateur padel players in Saudi Arabia, including injury characteristics, severity, and clinical outcomes (*N* = 187; Injured *n* = 28).

Variable	Category/Comparison	*n*	Value
Ankle sprain prevalence	Overall prevalence	28/187	14.97%
	95% CI	—	10.30 – 20.80%
Number of sprains	Mean ± SD	28	1.42 ± 0.68
Time since last injury (months)	Mean ± SD	28	5.83 ± 3.21
Injury laterality	Right	17	60.71%
	Left	7	25.00%
	Bilateral	4	14.29%
Mechanism of injury	Landing	16	57.14%
	Collision	5	17.86%
	Sudden direction change	4	14.29%
	Jumping/reaching	3	10.71%
Injury severity	Self-reported perceived mild	11	39.29%
	Self-reported perceived moderate	13	46.43%
	Self-reported perceived severe	4	14.29%
Time loss from sport (days)	Mean ± SD	28	3.12 ± 1.95
Pain score (0–10)	Mean ± SD	28	4.86 ± 1.72
Swelling	Yes	19	67.86%
	No	9	32.14%
Functional limitation	Yes	21	75.00%
	No	7	25.00%
Recurrent sprain	Yes	12	42.86%
	No	16	57.14%
Associated injuries	Knee	8	28.57%
	Lower back	5	17.86%
	Hip/groin	3	10.71%
Medical attention	Sought	0	0.00%
	Not sought	28	100.00%

Values are presented as mean ± SD or *n* (%). Prevalence was calculated with 95% confidence intervals and tested using a one-sample proportion test. CI, Confidence Interval; SD, Standard Deviation; *n*, Number of participants.

Participants with ankle sprain demonstrated a mean FADI score of 86.12 ± 8.74, indicating predominantly moderate functional disability, with 60.71% classified as having moderate disability and only 7.14% having severe disability (Table 3). The mean pain score was 4.68 ± 1.59, while perceived instability (60.71%), balance deficits (53.57%), strength deficits (50.00%), and recurrent sprains (42.86%) were common, despite a relatively short mean return-to-play time of 3.36 ± 1.88 days.

**Table 3:** Functional outcomes, disability profile, and association with preventive exercise among amateur padel players with ankle sprain in Saudi Arabia (*n* = 28).

Variable	Category/Comparison	*n*	Value	*p*-value
FADI total Score (0–104)	Mean ± SD	28	86.12 ± 8.74	—
	95% CI	—	82.79 – 89.45	—
	Range	—	70.00 – 102.00	—
Functional disability	Mild (90–99)	9	32.14%	—
	Moderate (70–89)	17	60.71%	—
	Severe (<70)	2	7.14%	—
Pain score (0–10)	Mean ± SD	28	4.68 ± 1.59	—
Perceived instability	Yes	17	60.71%	—
	No	11	39.29%	—
Giving way episodes (6 months)	Mean ± SD	28	2.07 ± 1.18	—
Return to play (days)	Mean ± SD	28	3.36 ± 1.88	—
Balance deficit	Yes	15	53.57%	—
	No	13	46.43%	—
Strength deficit	Yes	14	50.00%	—
	No	14	50.00%	—
Recurrent sprain	Yes	12	42.86%	—
	No	16	57.14%	—

Values are presented as mean ± SD or *n* (%). An independent-samples *t*-test was used to compare FADI scores between exercise groups. Pearson correlation was used to assess associations between exercise and functional outcomes. FADI; Foot and Ankle Disability Index; SD; Standard Deviation; CI; Confidence Interval; *n*; Number of participants.

Comparative analysis demonstrated that higher playing exposure was significantly associated with ankle sprain injuries, while most demographic and behavioral factors showed no significant differences between groups (Table 4). Players with ≥1 year of experience had markedly higher odds of injury (75.00% vs. 41.51%; OR = 4.23, *p* = 0.002), and those playing ≥2 sessions/week also showed increased risk (50.00% vs. 21.38%; OR = 3.68, *p* = 0.003). No significant differences were observed for age, BMI, sex, smoking, or chronic conditions (*p* > 0.05), indicating limited influence of baseline characteristics. Similarly, playing surface and warm-up practices were not associated with injury risk (*p* = 1.000), while non-padel footwear showed a non-significant trend toward higher risk (OR = 2.54, *p* = 0.081). Preventive behaviors, such as ankle support use and strengthening exercises, were also not significantly associated with injury occurrence, despite their higher prevalence among injured players.

**Table 4:** Comparison of demographic, clinical, playing-exposure, and modifiable behavioral risk factors between amateur padel players with and without ankle sprain injuries in Saudi Arabia.

Variable	Injured (*n* = 28)	Not injured (*n* = 159)	Effect estimate (95% CI)	*p*-value
Age, years	30.71 ± 6.34	29.63 ± 6.09	1.08 (-1.54 to 3.70)	0.409
Body mass index, kg/m^2^	26.41 ± 3.71	25.61 ± 3.82	0.80 (-0.75 to 2.35)	0.302
Male sex	24 (85.71%)	123 (77.36%)	1.76 (0.57 to 5.39)	0.457
Current smoking	10 (35.71%)	37 (23.27%)	1.83 (0.78 to 4.31)	0.245
Any chronic medical condition	18 (64.29%)	83 (52.20%)	1.65 (0.72 to 3.79)	0.328
Padel experience ≥1 year	21 (75.00%)	66 (41.51%)	4.23 (1.70 to 10.52)	0.002
Playing frequency ≥2 sessions/week	14 (50.00%)	34 (21.38%)	3.68 (1.60 to 8.45)	0.003
Artificial turf as usual playing surface	24 (85.71%)	134 (84.28%)	1.12 (0.36 to 3.51)	1.000
Non-padel-specific footwear	22 (78.57%)	94 (59.12%)	2.54 (0.97 to 6.60)	0.081
Inadequate warm-up (sometimes/never)	18 (64.29%)	104 (65.41%)	0.95 (0.41 to 2.20)	1.000
Never used ankle support	24 (85.71%)	121 (76.10%)	1.88 (0.62 to 5.77)	0.340
No ankle balance/strength exercises	18 (64.29%)	75 (47.17%)	2.02 (0.88 to 4.64)	0.143

Data are presented as mean ± SD or *n* (%). Effect estimates are shown as mean differences (95% CIs) for continuous variables and crude odds ratios (95% CIs) for categorical variables. Continuous variables were compared using independent-samples *t*-tests, and categorical variables were compared using Pearson's chi-square test or Fisher's exact test, as appropriate. CI, Confidence interval; SD, Standard deviation; kg/m^2^, kilograms per square meter; *n*, Number of participants; OR, Odds ratio.

**Figure 1:**
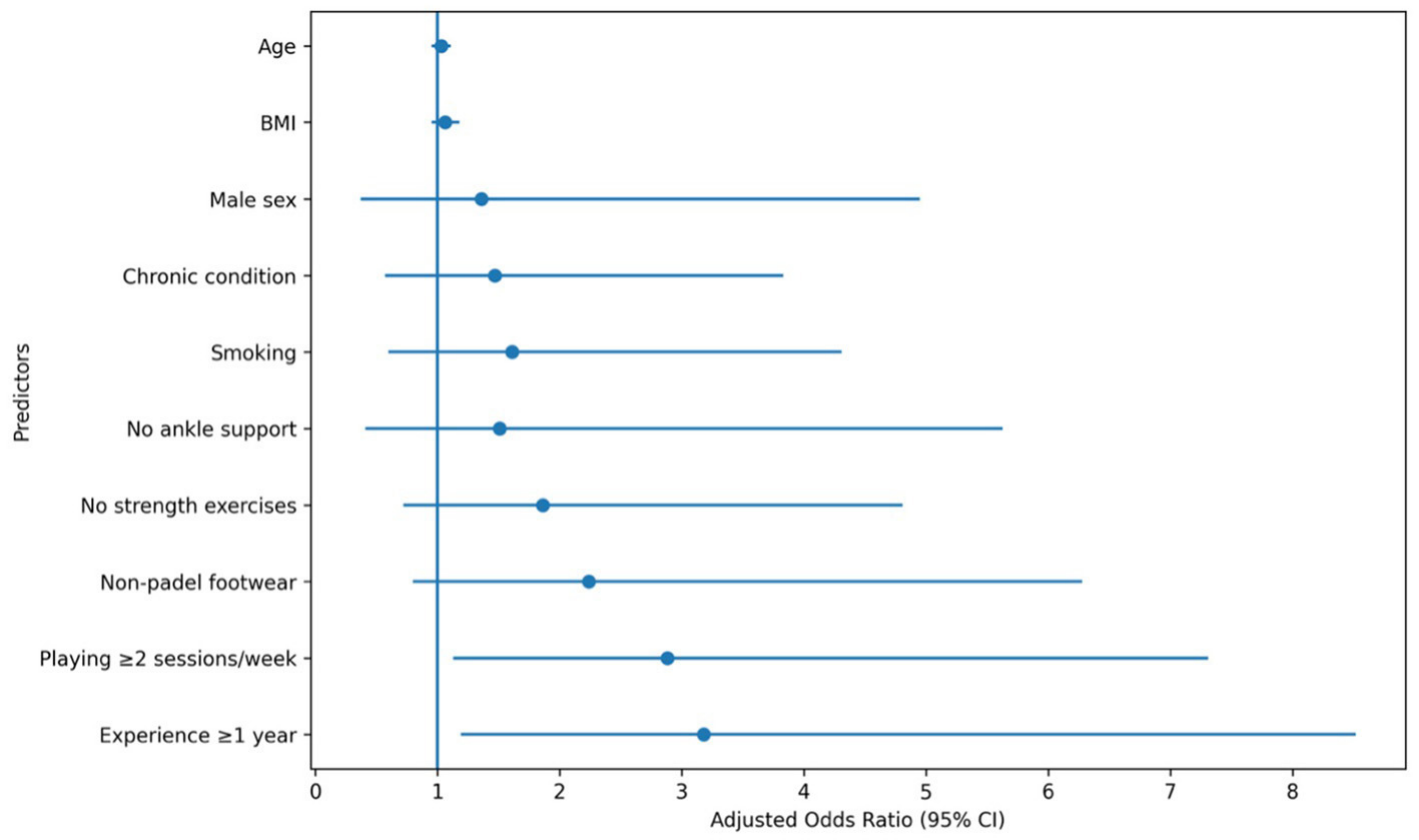
Adjusted odds ratios with 95% confidence intervals for predictors of ankle sprain among amateur padel players.

Multivariable analysis identified playing-exposure variables as the only independent predictors of ankle sprain, whereas demographic, clinical, and preventive factors were not significantly associated with ankle sprain (Table 5; Figure 1). Players with ≥1 year of experience had over 3-fold higher odds of injury (aOR = 3.18, 95% CI: 1.19–8.52, *p* = 0.021), and those playing ≥2 sessions/week also demonstrated increased risk (aOR = 2.88, 95% CI: 1.13–7.31, *p* = 0.026). No significant associations were observed for age, BMI, sex, smoking, or comorbidities (*p* > 0.05), indicating limited influence of baseline characteristics. Preventive behaviors, including warm-up, ankle support use, and strengthening exercises, were not independently associated with injury risk despite elevated odds ratios. The overall model demonstrated acceptable fit (Nagelkerke R^2^ = 0.23; *p* = 0.038) with good classification accuracy (85.60%).

**Table 5:** Multivariable logistic regression model for independent predictors of ankle sprain among amateur padel players in Saudi Arabia, adjusted for demographic, clinical, playing-exposure, footwear, and preventive behavioral factors (*N* = 187; Injured *n* = 28).

Predictor	Reference/Coding	Adjusted OR	95% CI	SE	*p*-value
Age, per 1-year increase	Continuous	1.03	0.95 to 1.11	0.04	0.505
Male sex	Female	1.36	0.37 to 4.95	0.66	0.642
Body mass index, per 1 kg/m^2^ increase	Continuous	1.06	0.95 to 1.18	0.05	0.276
Current smoking	No	1.61	0.60 to 4.31	0.50	0.344
Any chronic medical condition	No	1.47	0.57 to 3.83	0.48	0.428
Padel experience ≥1 year	<1 year	3.18	1.19 to 8.52	0.50	0.021
Playing frequency ≥2 sessions/week	1 session/week	2.88	1.13 to 7.31	0.46	0.026
Artificial turf as usual surface	Other surfaces	1.08	0.28 to 4.10	0.68	0.912
Non-padel-specific footwear	Padel-specific shoes	2.24	0.80 to 6.28	0.52	0.124
Inadequate warm-up (sometimes/never)	Always	0.91	0.35 to 2.39	0.49	0.853
Never used ankle support	Always/sometimes	1.51	0.41 to 5.63	0.67	0.542
No ankle balance/strength exercises	Yes/sometimes	1.86	0.72 to 4.81	0.48	0.203
**Predictor**	**Reference/Coding**	**Adjusted OR**	**95% CI**	**SE**	**p-value**
Age, per 1-year increase	Continuous	1.03	0.95 to 1.11	0.04	0.505
Male sex	Female	1.36	0.37 to 4.95	0.66	0.642
Body mass index, per 1 kg/m^2^ increase	Continuous	1.06	0.95 to 1.18	0.05	0.276
Current smoking	No	1.61	0.60 to 4.31	0.50	0.344
Any chronic medical condition	No	1.47	0.57 to 3.83	0.48	0.428
Padel experience ≥1 year	<1 year	3.18	1.19 to 8.52	0.50	0.021
Playing frequency ≥2 sessions/week	1 session/week	2.88	1.13 to 7.31	0.46	0.026
Artificial turf as usual surface	Other surfaces	1.08	0.28 to 4.10	0.68	0.912
Non-padel-specific footwear	Padel-specific shoes	2.24	0.80 to 6.28	0.52	0.124
Inadequate warm-up (sometimes/never)	Always	0.91	0.35 to 2.39	0.49	0.853
Never used ankle support	Always/sometimes	1.51	0.41 to 5.63	0.67	0.542
No ankle balance/strength exercises	Yes/sometimes	1.86	0.72 to 4.81	0.48	0.203

Model summary: binary logistic regression with ankle sprain status (injured vs not injured) as the dependent variable. The final model included 12 predictors; *N* = 187; events = 28; Cox & Snell R^2^ = 0.14; Nagelkerke R^2^ = 0.23; Hosmer–Lemeshow goodness-of-fit *p* = 0.741; overall model χ^2^(12) = 21.96, *p* = 0.038; classification accuracy = 85.60%. OR, odds ratio; CI, confidence interval; SE, standard error; kg/m^2^, kilograms per square meter; R^2^, coefficient of determination; χ^2^, chi-square; N, number of participants.

**Figure 2:**
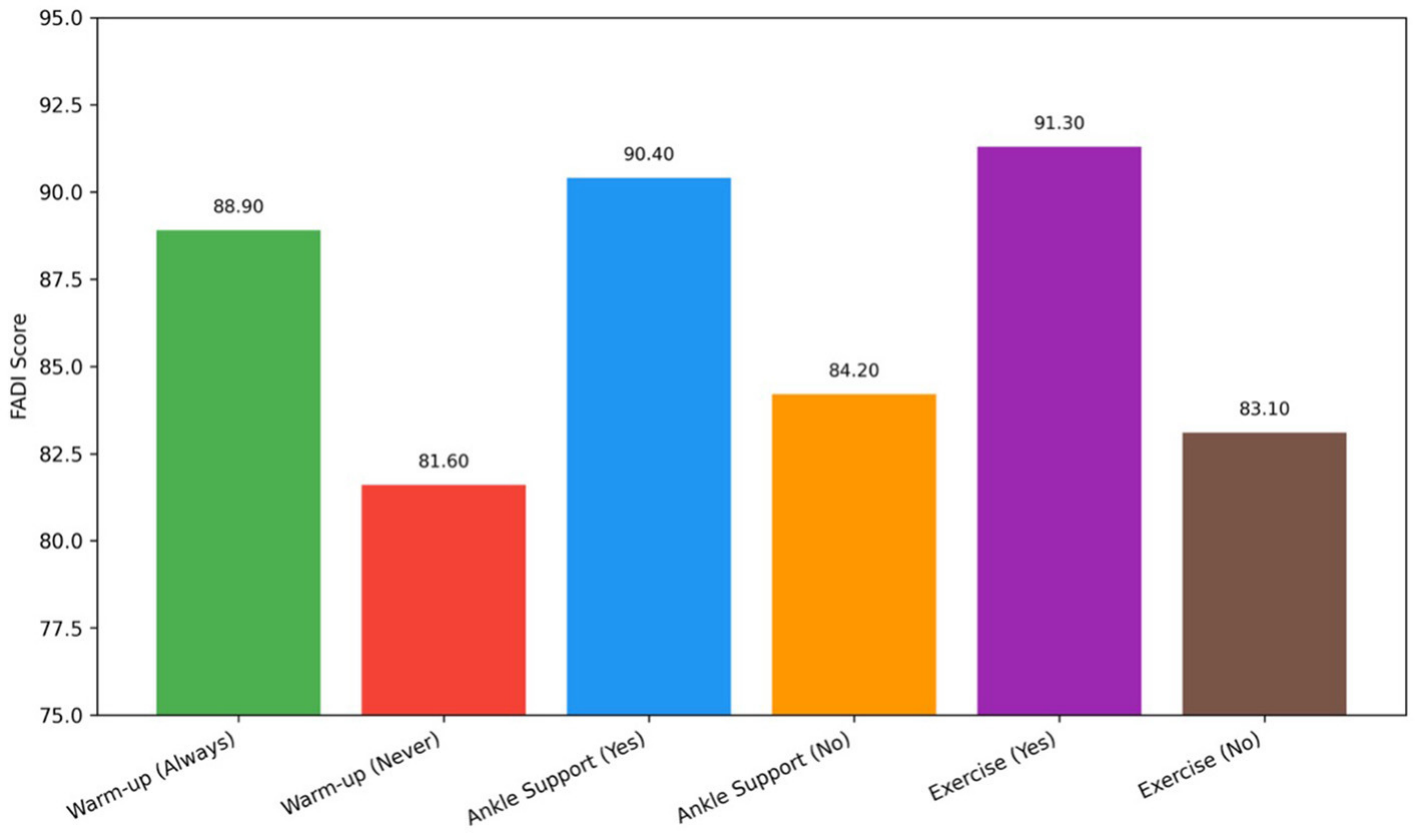
Association between preventive behaviors and functional outcomes (FADI Scores) among amateur padel players with ankle sprain.

Preventive behaviors were significantly associated with improved functional outcomes, with consistent trends observed across all modalities (Table 6; Figure 2). Players who always performed warm-up demonstrated higher FADI scores compared to those who never did (88.90 ± 8.10 vs. 81.60 ± 9.20; Δ = 7.30, *p* = 0.041; η^2^ = 0.18). Similarly, ankle support use was linked to better function (90.40 ± 7.95 vs. 84.20 ± 8.70; Δ = 6.20, *p* = 0.027). The strongest association was observed for balance and strengthening exercises, with active participants showing higher functional scores (91.30 ± 7.60 vs. 83.10 ± 8.90; Δ = 8.20, *p* = 0.008; η^2^ = 0.24). Additionally, a moderate positive correlation was found between exercise engagement and FADI scores (*r* = 0.34, *p* = 0.028), indicating a meaningful relationship between preventive behaviors and functional recovery.

**Table 6:** Association between preventive behaviors and functional outcomes (FADI Scores) among amateur padel players with ankle sprain in Saudi Arabia, including effect sizes, mean differences, and comparative analyses (*n* = 28).

Preventive behavior	Category	*n*	FADI score (Mean ±SD)	Effect size/mean difference (95% CI)	*p*-value
Warm-up	Always	10	88.90 ± 8.10		
	Sometimes	11	85.70 ± 8.85		
	Never	7	81.60 ± 9.20	η^2^ = 0.18; Δ (always–never) =7.30 (0.50–14.10)	0.041
Ankle support	Always/Sometimes	9	90.40 ± 7.95	6.20 (0.80–11.60)	0.027
	Never	19	84.20 ± 8.70		
Balance/Strength exercises	Yes	8	91.30 ± 7.60		
	Sometimes	9	87.20 ± 8.30		
	No	11	83.10 ± 8.90	η^2^ = 0.24; Δ (yes–no) =8.20 (1.20–15.20)	0.008
Correlation: exercise vs.FADI	Pearson r	28	0.34	–	0.028

Values are presented as mean ± SD. One-way ANOVA was used for multi-group comparisons, with η^2^ as the effect size, and pairwise mean differences (Δ) with 95% CIs are reported for key contrasts. An independent samples *t*-test was used for binary comparisons. Pearson correlation was used to assess associations between exercise and functional outcomes. FADI; Foot and Ankle Disability Index; SD; Standard Deviation; η^2^; Eta squared; CI; Confidence Interval; Δ; Mean difference; *n*; Number of participants.

## 4 Discussion

This study aimed to determine the prevalence and clinical profile of ankle sprain injuries among amateur padel players in Saudi Arabia, identify associated risk factors, and evaluate the association between preventive behaviors and functional outcomes. The findings demonstrated that ankle sprains are relatively common in this population and are characterized by notable functional impairment and persistent symptoms despite short return-to-play durations. Increased playing exposure, particularly in terms of experience and frequency, emerged as the primary factor associated with injury occurrence, whereas demographic and general health characteristics showed limited influence. In contrast, preventive behaviors were not independently associated with reduced injury risk but were consistently linked to improved functional outcomes, indicating their greater relevance to recovery than to primary prevention. Collectively, these results highlight the dual burden of injury occurrence and residual dysfunction, while emphasizing the importance of targeted rehabilitation-focused strategies in amateur padel players.

Ankle sprain injuries in amateur padel players were found to present with a distinct clinical profile characterized by moderate disability, persistent symptoms, and limited healthcare utilization, which may be explained by both sport-specific biomechanical demands and behavioral factors (18). Padel involves rapid lateral movements, frequent deceleration, and repetitive jumping and landing, which increase mechanical stress on the lateral ankle complex and predispose players to ligamentous injuries, particularly during landing phases (8). This is consistent with findings from Alhammad et al. (21) and Belmar-Arriagada et al. (14), who reported that the dynamic and multidirectional nature of padel substantially elevates the risk of lower-limb injury, particularly at the ankle. Additionally, the high prevalence of functional limitations and recurrent symptoms may be attributed to inadequate injury management and premature return to play, as none of the injured players sought medical attention (1). Similar patterns have been described by Rowe et al. (22) and Roch et al. (23), who highlighted that insufficient rehabilitation and early return to activity contribute to chronic ankle instability and long-term functional deficits. Together, these factors explain the observed combination of relatively short time loss with ongoing impairment and recurrent injury episodes (24).

Increased playing exposure emerged as the primary factor associated with ankle sprain occurrence, whereas preventive behaviors were more strongly related to functional recovery than to injury prevention, reflecting distinct underlying mechanisms (18). Higher exposure, in terms of playing experience and frequency, likely increases cumulative load and repeated microtrauma to the ankle joint, thereby elevating injury risk over time (25). This aligns with previous research by Merza et al. (25) and Schmitt et al. (26), which demonstrated that greater sports participation and exposure are key determinants of injury incidence across athletic populations (25). In contrast, preventive strategies such as warm-up routines, ankle support, and strengthening exercises were not independently associated with reduced injury occurrence but were linked to improved functional outcomes (27). This may be explained by their role in enhancing neuromuscular control, proprioception, and joint stability following injury, rather than by preventing the initial injury (28). Supporting this, Naderifar et al. (28) and Karami et al. (29) reported that balance and strengthening interventions are effective in improving post-injury function and reducing residual instability, although their effect on primary injury prevention is less consistent. These findings collectively suggest that while exposure-related factors drive injury risk, rehabilitation-focused behaviors play a critical role in optimizing recovery and functional performance (30).

The observed between-group differences in FADI scores ranged from 6.29 to 8.20 points. Published evidence suggests that the minimal clinically important difference (MCID) for the FADI is approximately 8 points (31, 32). Accordingly, larger observed differences may approach or reach clinical importance, whereas smaller differences should be interpreted with caution despite their statistical significance. These findings indicate that preventive behaviors may be associated with functionally meaningful improvements in some participants; however, confirmation in prospective studies is warranted.

## 4.1 Clinical significance

This study provides clinically relevant evidence that ankle sprain injuries among amateur padel players are not only relatively prevalent but are also associated with persistent functional impairments despite minimal time loss from sport. The findings highlight a critical mismatch between perceived recovery and actual functional status, as a substantial proportion of players demonstrated instability, recurrent sprains, and residual deficits without seeking medical care. Importantly, identifying playing exposure as the primary factor associated with injury risk underscores the need for targeted load management and structured participation guidelines in this population. Furthermore, the demonstrated association between preventive behaviors and improved functional outcomes underscores the clinical value of incorporating structured rehabilitation strategies, such as balance and strengthening exercises, into routine training to enhance recovery and reduce long-term disability.

## 4.2 Limitations of the study and areas of future research

Several limitations should be considered when interpreting these findings. The cross-sectional design precludes causal inference and limits the ability to establish temporal relationships between exposure, injury, and recovery outcomes. Data were collected exclusively through self-reported questionnaires, without objective verification by medical record review or clinical examination, introducing the potential for recall bias, reporting bias, and injury misclassification. Because ankle sprain history was self-reported, mild injuries may have been underreported, whereas recurrent injuries may have been overreported. The predefined 12-month recall period may have reduced reporting accuracy, particularly for less severe injuries. Recall reliability was not formally assessed, and a *post-hoc* sensitivity analysis using a shorter recall period was not feasible because such data were not collected. Participant recruitment through convenience sampling and social media platforms may also have introduced selection bias by preferentially enrolling individuals who were more active, engaged with padel, or health-conscious than the broader amateur padel population. Because participation was voluntary, the response rate could not be determined, and the characteristics of participants and non-participants could not be compared, thereby limiting the representativeness and generalizability of the findings. Generalizability is further constrained by the inclusion of amateur padel players from a single geographic region. Moreover, the absence of objective clinical and biomechanical assessments limited the comprehensive evaluation of injury severity and functional impairment, while self-reported pain scores may have been influenced by individual differences in pain perception and thresholds. Residual confounding is also possible because several potentially important variables, including previous musculoskeletal injuries other than ankle sprains, baseline lower extremity muscle strength, sport-specific training volume (e.g., frequency of matches or training sessions), and BMI categorized according to established clinical classifications, were not assessed and may have influenced both injury risk and functional outcomes. Future prospective longitudinal studies incorporating objective clinical verification, biomechanical and functional assessments, shorter follow-up intervals, and comprehensive adjustment for relevant confounding variables are needed to better establish causal relationships and evaluate the effectiveness of targeted injury prevention and rehabilitation strategies in reducing ankle sprain incidence and long-term functional deficits among padel players.

## 5 Conclusions

Ankle sprain injuries among amateur padel players in Saudi Arabia are relatively common and are characterized by moderate functional impairment, persistent symptoms, and a high prevalence of recurrent instability despite short return-to-play durations. Increased playing exposure, particularly in terms of experience and frequency, was identified as the primary factor associated with injury occurrence, while demographic and general health characteristics showed limited influence. Preventive behaviors were not independently associated with injury risk but were consistently linked to improved functional outcomes, indicating their importance in recovery rather than primary prevention. These findings underscore the need for improved injury management strategies, greater emphasis on rehabilitation-focused interventions, and increased awareness of functional deficits following ankle sprain in this population.

## Data Availability

The original contributions presented in the study are included in the article/supplementary material, further inquiries can be directed to the corresponding author.
